# Strain Diversity of *Mycobacterium tuberculosis* Isolates from Pulmonary Tuberculosis Patients in Afar Pastoral Region of Ethiopia

**DOI:** 10.1155/2014/238532

**Published:** 2014-03-06

**Authors:** Mulugeta Belay, Gobena Ameni, Gunnar Bjune, David Couvin, Nalin Rastogi, Fekadu Abebe

**Affiliations:** ^1^Aklilu Lemma Institute of Pathobiology, Addis Ababa University, P.O. Box 1176, Addis Ababa, Ethiopia; ^2^Section for International Health, Department of Community Medicine, Institute for Health and Society, University of Oslo, P.O. Box 1130, Blindern, 0318 Oslo, Norway; ^3^WHO Supranational TB Reference Laboratory, TB & Mycobacteria Unit, Institut Pasteur de la Guadeloupe, 97183 Abymes, France

## Abstract

Data on genotypic diversity of *Mycobacterium tuberculosis* complex (MTBC) is important to understand its epidemiology, human adaptation, clinical phenotypes, and drug resistance. This study aimed to characterize MTBC clinical isolates circulating in a predominantly pastoralist area in Ethiopia, a country where tuberculosis is the second leading cause of mortality. Culture of sputum samples collected from a total of 325 pulmonary TB suspects was done to isolate MTBC. Spoligotyping was used to characterize 105 isolates from culture positive slopes and the result was compared with an international database. Forty-four spoligotype patterns were observed to correspond to 35 shared-types (SITs) containing 96 isolates and 9 orphan patterns; 27 SITs containing 83 isolates matched a preexisting shared-type in the database, whereas 8 SITs (*n* = 13 isolates) were newly created. A total of 19 SITs containing 80 isolates were clustered within this study (overall clustering of 76.19%). Three dominant lineages (T, CAS, and Manu) accounted for 76.19% of the isolates. SIT149/T3-ETH was one of the two most dominant sublineages. Unlike previous reports, we show that Manu lineage strains not only constitute a dominant lineage, but are also associated with HIV infection in Afar region of Ethiopia. The high level of clustering suggests the presence of recent transmission that should be further studied using additional genotyping markers.

## 1. Introduction

Tuberculosis (TB) is among the 10 major causes of death [1] and it is only outranked by HIV/AIDS among infectious causes of death worldwide [2]. Despite the global effort towards controlling TB for the last 20 years, the burden remains high with over 8.7 million incident cases and 1.4 million deaths in 2011 [3]. The HIV epidemics and the spread of MDR-TB are among the major obstacles for the successful control of TB and, in this regard, Africa is lagging behind in achieving to halve the 1990s mortality by 2015 [3].

Ethiopia is among the 22 high TB as well as the 27 high MDR-TB burden countries [3]. According to the Ministry of Health, TB is the second leading cause of death [4]. Apart from other factors, the HIV epidemics and the emergence of MDR-TB have contributed to the high TB burden in the country. According to the recent national TB prevalence survey, the prevalence of smear positive pulmonary TB is highest among pastoral communities [5].

Genotypic variations among* M. tuberculosis* strains as well as the existence of human genetic polymorphism linked to TB have resulted in the changing relationship between* M. tuberculosis* and the human host. Such changes have complicated TB control efforts [6]. Data on strain diversity of mycobacterial isolates is important to understand the transmission dynamics and phylogeographical distribution of dominant circulating strains of* M. tuberculosis*.

This study was part of a major project on molecular epidemiology, clinical epidemiology, and immunology of TB in a pastoral community and their livestock in Ethiopia. Some studies have reported the dominant strains among human isolates in central highlands of Ethiopia [7–11]; besides, strains isolated from goats and camels have been reported from Afar region recently [12, 13]. This study reports strains of* M. tuberculosis* isolates circulating in the Afar pastoral communities.

## 2. Methods

### 2.1. Study Area

The study area has been described elsewhere [14]. Briefly, the study was conducted in two public (Awash Health Centre and Dubti Hospital) health facilities in Afar region and three private (Selam hospital, Bati Hospital, and Amir Higher Clinic) health facilities in Dessie town. The reason for including these private health facilities is because they provide diagnostic services for a substantial number of TB patients coming from Afar region.

### 2.2. Study Design and Study Participants

A health facility-based cross-sectional study was conducted between September 2009 and March 2010. A total of 325 pulmonary TB suspects (≥18 years of age) who were residents of Afar region during the study period and came to the selected health facilities with cough lasting more than 2 weeks were included consecutively. TB patients who were already taking anti-TB drugs were excluded.

### 2.3. Data Collection

A semi-structured, pretested questionnaire was used to collect data on basic sociodemographic characteristics as well as clinically relevant symptoms. Three sputum samples (spot-morning-spot) were collected and smear microscopic examination for AFB was done at the respective health facilities according to the national TB and Leprosy control programme guideline [15]. The rest of the sputa were stored at +4°C and transported to Aklilu Lemma Institute of Pathobiology (ALIPB), Addis Ababa University, within 1 week. Participants were tested for HIV according to the national guideline [16].

### 2.4. Mycobacterial Culture

The three sputa from each participant were pooled at ALIPB laboratory; culture was done according to the WHO guideline [17]. Briefly, sputum samples were homogenized and decontaminated with equal volume of 4% NaOH and shaken for 15 minutes at room temperature. Subsequently, it was centrifuged at 3000 rpm for 15 minutes. After the supernatant was poured off, the sediment was neutralized with 2 N HCL and 2–4 loopfuls of the centrifuged sediment were inoculated into four slopes of LJ medium. Inspection of media was done every week for growth until 8 weeks. Based on colony morphology and smear microscopy, those with growth were identified and two colonies were transferred to eppendorf tube with 300 *μ*L distilled water. Mycobacterial genomic DNA was extracted by heating isolates at 80°C for 60 minutes. Heat-killed isolates were stored at −20°C until spoligotyping was done.

### 2.5. Spoligotyping and Database Comparison

Spoligotyping of 105 isolates was performed using a commercially available kit from Ocimum Biosolutions Company, Iisselstein, The Netherlands, according to the company's instructions and as described previously [9, 18]. Briefly, the direct repeat (DR) region was amplified by a Thermal Cycler PCR machine (VWR International) using oligonucleotides and primers derived from this region. The amplified product was hybridized to a set of 43 immobilized oligonucleotides, each corresponding to one of the unique spacer. Hybridized DNA was detected by chemiluminescence method (Amersham Biosciences, Little Chalfont, UK) and by exposure to X-ray film (Hyperfilm ECL, Amersham Biosciences), as specified by the manufacturer. The hybridization patterns were converted into binary and octal formats and compared with previously reported strains in the in-house SITVIT2 proprietary database of Institut Pasteur de la Guadeloupe, which is an updated version of the recently released SITVITWEB database [19]. In this database, a Spoligotype International Type (SIT) designates a pattern shared by 2 or more patient isolates, whereas “orphan” represents a pattern reported for a single isolate. At the time of this comparison, SITVIT2 contained genotyping data on more than 110,000 MTBC clinical isolates from 160 countries of patient origin, and >3500 SITs. Major phylogenetic clades were essentially assigned according to signatures provided earlier [19]; these included specific signatures for various MTBC members as well as rules defining major lineages/sublineages for* M. tuberculosis* sensu stricto. The latter included the Beijing clade, the Central-Asian (CAS) clade and its 2 sublineages, the East-African-Indian (EAI) clade with its 9 sublineages, the Haarlem clade and its 3 sublineages, the Latin-American-Mediterranean (LAM) clade and its 10 sublineages, the “Manu” family and its 3 sublineages, the IS*6110*-low banding X clade and its 3 sublineages, and the ill-defined T clade and its 5 sublineages. The high phylogeographical specificity of LAM10-CAM prototype SIT61 for Cameroon has led to its designation as the Cameroon lineage [20], whereas LAM7-TUR lineage was tentatively reclassified as the Turkey lineage [21].

We also studied the worldwide distribution of all major spoligotyping clusters in our study (clusters containing 3 or more isolates) by interrogating the database for their distribution in macrogeographical regions and subregions according to the United Nations (http://unstats.un.org/unsd/methods/m49/m49regin.htm; for further details please refer to the footnote of Table 3). Lastly, the distribution of predominant spoligotype patterns in this study (*n* = 105 strains) was further compared to all other strains reported from Ethiopia in the international database (*n* = 1507 strains excluding the present study) as well as the five African subregions (*n* = 13656 strains), and the neighboring Western Asian countries (*n* = 4790 strains; for further details please refer to the footnote of Table 4).

Minimum spanning trees (MSTs) illustrating evolutionary relationships between the* M. tuberculosis* spoligotypes and various sociodemographic variables were constructed using BioNumerics software version 6.6 (Applied Maths, Sint-Martens-Latem, Belgium). MST is an undirected network in which all of the samples are linked together with the fewest possible linkages between nearest neighbors. Lastly, Spoligoforest trees which represent another way to illustrate probable strain evolutionary relationships between spoligotypes were drawn [22, 23] using spolTools online utilities available through http://www.emi.unsw.edu.au/spolTools. As opposed to the MSTs, the spoligoforest trees are directed and only evolve by loss of spacers. GraphViz software (http://www.graphviz.org/ [24]) was used to color the strains based on their lineages on the spoligoforest trees.

### 2.6. Ethics Statement

The study has been ethically cleared by the Norwegian Ethics Committee and the Ethiopian National Ethics Committee. Written informed consent was obtained from each study participant.

### 2.7. Statistical Analysis

Chi-square test using STATA software version 12 was used to evaluate whether or not significant association may exist between lineages or SITs with sociodemographic and epidemiological characteristics. Pearson's Chi-square test was used when more than 80% of data had an expected value greater than 5 and Fisher's Exact Test for remaining data with smaller expected values (at least 20% of data having values less than 5). *P* values less than 0.05 were considered as statistically significant.

## 3. Results

### 3.1. Demographic Characteristics of Study Participants

Among 325 pulmonary TB suspects, a total of 105 patients were confirmed to have culture positive pulmonary TB and included for further analysis. The median age of patients was 29 (IQR: 22.5–40.0) years and the majority were males with a male to female ratio of 1.92. Thirty-nine (37%) of culture confirmed TB patients were positive for acid fast bacilli on sputum smear examination. Besides, HIV result was available for 95 of the patients and 39% of them were found to be HIV co-infected. The majority (77.1%) of the study participants were new cases of pulmonary TB. Pastoralists represented 42.9% of the study participants. The proportion of HIV positives among ethnic Afar (8.7%) was significantly lower compared to other ethnic groups (28.0–47.5%) (*P* < 0.001).

### 3.2. Genetic Diversity and Distribution of Isolates

Overall, 103 (98.1%) isolates had spoligotypes characteristic of* M. tuberculosis* complex and 2 (1.9%) isolates had spoligotypes characteristic of* M. bovis*. A total of 9 isolates showed orphan patterns (Table 1), whereas 16 strains were unique making the total number of unclustered isolates to be 25 (23.81%). On the other hand, 19 SITs containing 80 isolates (76.19%) were clustered within this study (2 to 10 isolates per cluster). Besides, a total of 44 spoligotype patterns were observed and, therefore, the overall diversity of the isolates was 41.9%. A total of 27 SITs containing 83 isolates matched a preexisting shared-type in the SITVIT2 database, whereas 8 SITs (*n* = 13 isolates) did not match any of the previous isolates in the database and hence were newly created (Table 2).

The majority (76.19%) of the isolates belonged to the following three dominant lineages: T (*n* = 43, 40.95%), Manu (*n* = 20, 19.05%), and CAS (*n* = 17, 16.19%). The other minor lineages in this study include X (*n* = 6, 5.71%), Haarlem (*n* = 4, 3.81%), EAI (*n* = 4, 3.81%), LAM (*n* = 3, 2.86%), BOV (*n* = 2, 1.90%), AFRI (*n* = 1, 0.95%), Turkey (*n* = 1, 0.95%), and S (*n* = 1, 0.95%) (Table 2). Three isolates (2.86%) had patterns with signatures that do not belong to any of the major lineages described in the database and hence labelled as “unknown” (Tables 1 and 2). No Beijing strains were identified in this study.

The dominant SITs in this study included SIT 149 of the T3-ETH lineage, SIT 37 of the T3 lineage, SIT 53 of the T1 lineage, and SIT 54 of the Manu2 lineage all together accounting for 36.2% of the isolates (Table 3). On comparing the dominant isolates from this study with the isolates in the SITVIT2 database from Ethiopia, the five African regions, and neighboring Western Asia, statistically significant differences were observed (Table 4). Significantly higher proportions of T2 (*P* < 0.01), X1 (*P* < 0.01) and H (*P* = 0.024) lineages/sublineages were found among isolates in this study compared to their representations in the SITVIT2 database from Ethiopia. Notably, the Manu lineage was significantly higher compared to previous isolates from Africa and neighboring Western Asia (*P* < 0.01). Besides, this lineage has not been reported to SITVIT2 database from Ethiopia so far. On the other hand, there was no significant difference in the distribution of T1, T3, T3-ETH, and CAS1-Delhi lineages compared to previous isolates from Ethiopia (*P* > 0.05).

**Figure 1:**
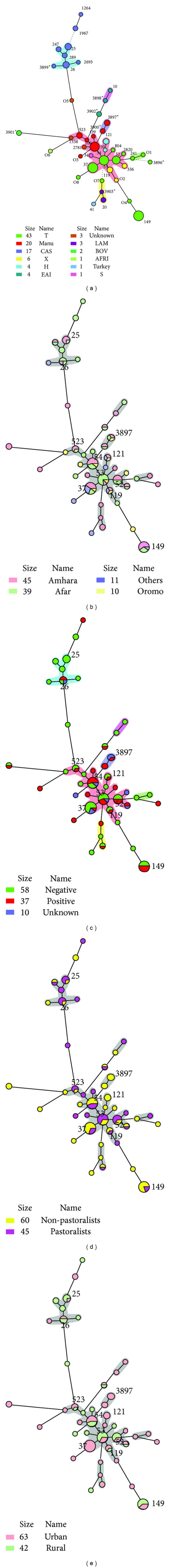
A minimum spanning tree (MST) illustrating evolutionary relationships between the* M. tuberculosis* spoligotypes (a) and various parameters studied such as ethnicity of patients (b), HIV serology (c), occupation (d), and residence (e). Note that the MSTs were constructed using BioNumerics 6.6 on all spoligotypes (*n* = 105). The phylogenetic tree connects each pattern based on degree of changes required to go from one allele to another. The structure of the tree is represented by branches (continuous versus dashed and dotted lines) and circles representing each individual pattern. Note that the length of the branches represents the distance between patterns while the complexity of the lines (continuous, gray dashed, and gray dotted) denotes the number of allele/spacer changes between two patterns: solid lines, 1 or 2 or 3 changes (thicker ones indicate a single change, while the thinner ones indicate 2 or 3 changes); grey dashed lines represent 4 changes; and dotted lines represent 5 or more changes. The size of the circle is proportional to the total number of isolates in our study, illustrating unique isolates (smaller nodes) versus clustered isolates (bigger nodes). The colour of the circles indicates the phylogenetic lineage to which the specific pattern belongs.

**Figure 2:**
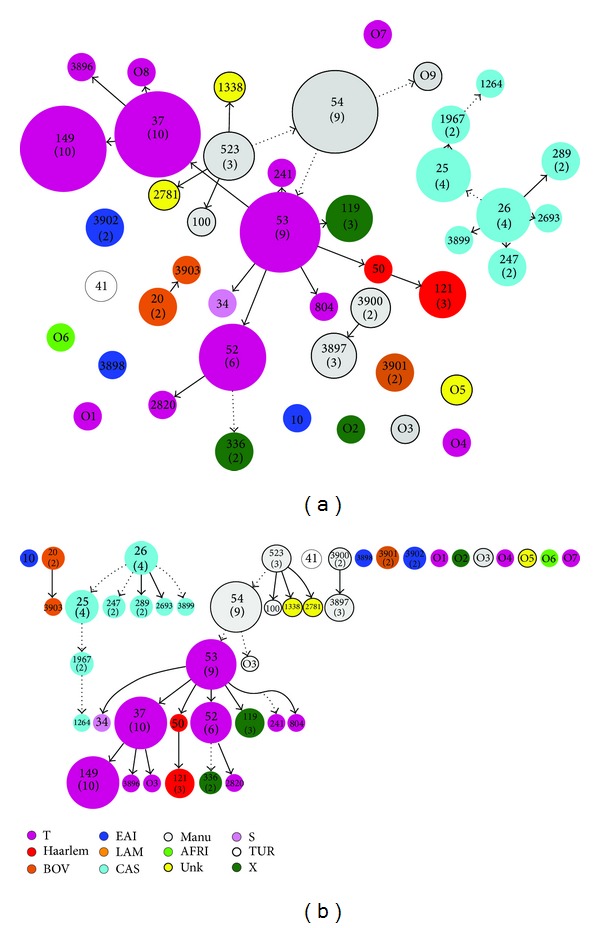
Spoligoforest trees were drawn using the spoligotyping data (*n* = 105 isolates) and the SpolTools software ([22]; available through on http://www.emi.unsw.edu.au/spolTools) and reshaped and colored manually by the GraphViz software (http://www.graphviz.org/; [24]). Two different trees were drawn using the Fruchterman Reingold algorithm (a) and the Hierarchical Layout (b). Note that the trees illustrate each spoligotype pattern from our study by a node with area size being proportional to the total number of isolates with that specific pattern. Changes (loss of spacers) are represented by directed edges between nodes, with the arrowheads pointing to descendant spoligotypes. The heuristic used selects a single inbound edge with a maximum weight using a Zipf model. The significance of the edges is the same for Hierarchical Layout and Fruchterman Reingold trees. Solid black lines link patterns that are very similar, that is, loss of one spacer only (maximum weigh being 1.0), while dashed lines represent links of weight comprised between 0.5 and 1 and dotted lines a weight less than 0.5. In both trees, one can denote that SIT149/T3-ETH and SIT37/T3 are the biggest nodes (*n* = 10), followed by SIT53/T1 and SIT54/Manu2 (*n* = 9), SIT52/T2 (*n* = 6) and SIT25, and SIT26/CAS1-Delhi (*n* = 4), which are the other predominant patterns in our study.

A minimum spanning tree (MST) illustrating evolutionary relationships among the strains has been constructed (Figure 1). In this evolutionary tree, MANU strains were closest to the central node of the unrooted tree which is represented by SIT53, whereas CAS strains are further away from the central node of the tree. On spoligoforest trees (Figure 2), SIT149/T3-ETH and SIT37/T3 are the biggest nodes (each with 10 isolates), followed by SIT53/T1 and SIT54/Manu2 (each with 9 isolates); SIT52/T2 (*n* = 6), SIT25, and SIT26/CAS1-Delhi (each with 4 isolates) are the other dominant isolates in our study.

On investigating the association between strains and HIV co-infection, strains belonging to Manu2 lineage were significantly associated with HIV infection compared to strains belonging to CAS lineage (Fisher's Exact Test, *P* = 0.019). However, strain clustering was not significantly associated with HIV infection and other sociodemographic characteristics of the study participants.

## 4. Discussion

Analysis of strain diversity and comparison with international database is key to have an insight into the global distribution of* M. tuberculosis* strains. In this regard, we describe the strain diversity of* M. tuberculosis* complex among clinical isolates of pulmonary TB patients from a predominantly pastoralist area in Northeast Ethiopia and compared the strains with SITVIT2 database strains.

Among 105 isolates, the majority (98.1%) of the isolates were identified as* M. tuberculosis* complex with only 2 (1.9%) isolates being* M. bovis*. This finding is in agreement with previous reports from Ethiopia [25, 26]. Unlike a previous study from Ethiopia [27] which reported a significant contribution (17%) of bovine TB among TB lymphadenitis patients, the contribution of* M. bovis* to human TB seems to be minimal and mainly restricted to the pastoral communities as supported by current evidence [25, 26].

In our study, the overall clustering was 76.19% indicating a high rate of recent transmission in the study area. Although this high level of spoligotyping based clustering should be further studied using additional genotyping markers, it is comparable with a previous study from Addis Ababa [8]. The recent national TB prevalence survey also argues for an ongoing TB epidemic mainly affecting the young probably indicating a defect in the TB control programme [28]. Besides, we have previously reported [29] that TB patients in Afar region suffered long diagnostic delays contributing to continued transmission with strain circulation.

Three dominant lineages were identified in this study and in agreement with previous reports [8, 9], the ill-defined T-lineage is the most dominant lineage accounting for 40.95% of the isolates. Among the T-lineages, T3-ETH is the most common lineage accounting for 25.6% of the T-lineage in this study. Similarly, previous studies from Ethiopia [8, 9, 11] reported a high proportion of T3-ETH among isolates indicating that this lineage is one of the dominant lineages in the country. CAS-Delhi lineage is the other dominant lineage in this study accounting for 16.19% of the isolates. In agreement with our finding, this lineage has been reported in previous studies as one of the dominant lineages in Ethiopia [7–9, 11] as well as in neighboring Djibouti [30]. EAI lineage was also isolated in smaller proportion in the study area. Both CAS and EAI are mainly prevalent in Middle East and Central Asia (Table 3). Two hypotheses could explain the presence of these lineages in Ethiopia as well: (i) it could have emerged in Ethiopia and migrated to Middle East and Central Asia, a hypothesis in agreement with the suggestion that East Africa is the cradle of* M. tuberculosis* complex species [31]; this is supported by a recent evidence [32] that MTBC co-evolved with the modern human host and migrated from Africa (particularly East Africa) to Asia and other parts of the world; (ii) alternatively, it could be due to migration of this lineage from Middle East and Central Asia to Ethiopia due to the recent human migrations from these areas to Ethiopia as suggested by a previous study [7].

Interestingly, Manu is one of the dominant lineages in our study contributing to 19.05% of the isolates. In agreement with this, investigators from Egypt reported a high proportion (27%) of Manu among their isolates [33]. In Ethiopia, only one study [9] from the Central highlands reported SIT 54 contributing to 8.33% of the isolates which is similar to our finding for SIT 54. However, Manu has not been reported from Ethiopia in other previous studies [7, 8, 11] as well as in the SITVIT2 database in which SIT 54 (Manu2) has been mainly reported from South and East Asia, Middle East including Egypt, and America. Furthermore, a recent study on a relatively large number of isolates mainly from Djibuti [34] did not find Manu lineage. The diverse distribution of strains in the study areas included in Ethiopia so far implies the need to map the distribution of* Mycobacterium tuberculosis* in the country. Interestingly, Manu2 lineage was found to be significantly associated with HIV infection although the reason for such association is not apparent from the current study.

Regarding the evolutionary relationship among strains, as opposed to CAS strains, the Manu strains in the MST tree are the ones closest to the dominant T lineage as reported before [33]. Besides, the T1 strain represented by its prototype SIT53 constitutes the central node of this unrooted tree and SIT 52 of T2 and SIT 37 of T3 are the other big nodes closest to the central node (Figure 1(a)). SIT149 (T3-ETH), which is the commonest lineage in Ethiopia, is further from the central node.

In conclusion, this study identified the presence of high level of clustering suggesting the presence of recent transmission in the study area. Nonetheless, one limitation of the present study is the fact that Mycobacterial interspersed repetitive units-variable number of tandem repeats (MIRU-VNTR) [35, 36] that allows splitting certain spoligotyping defined clusters in smaller subclusters [37] could not be used in the present study. Considering a recent study showing that some of the Manu lineage strains could result from mixed or polyclonal infections [38], and seeing the higher than usual proportion of Manu lineage strains in our study, it might be important to systematically perform MIRU typing of all spoligotyping clusters in future studies in the Afar pastoral region. We suggest that a nationwide study aiming to map the population structure of* M. tuberculosis* complex should be planned using spoligotyping, MIRU-VNTRs, and SNP in Ethiopia.

## Figures and Tables

**Table 1:** Orphan strains (*n* = 9) and corresponding spoligotyping defined lineages/sublineages recorded among a total of 105 *M. tuberculosis* strains isolated in Afar Region, Northeast Ethiopia.

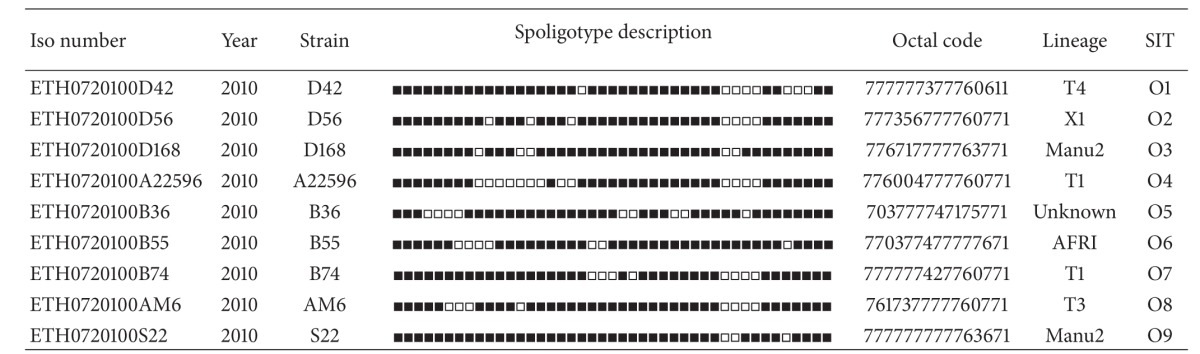

*Lineage designations for orphan patterns were done manually as expert-based interpretations using revised SpolDB4 rules.

**Table 2:** Description of 35 shared-types (SITs; *n* = 96 isolates) and corresponding spoligotyping defined lineages/sublineages starting from a total of 105 *M. tuberculosis* strains isolated in Addis Ababa (Ethiopia).

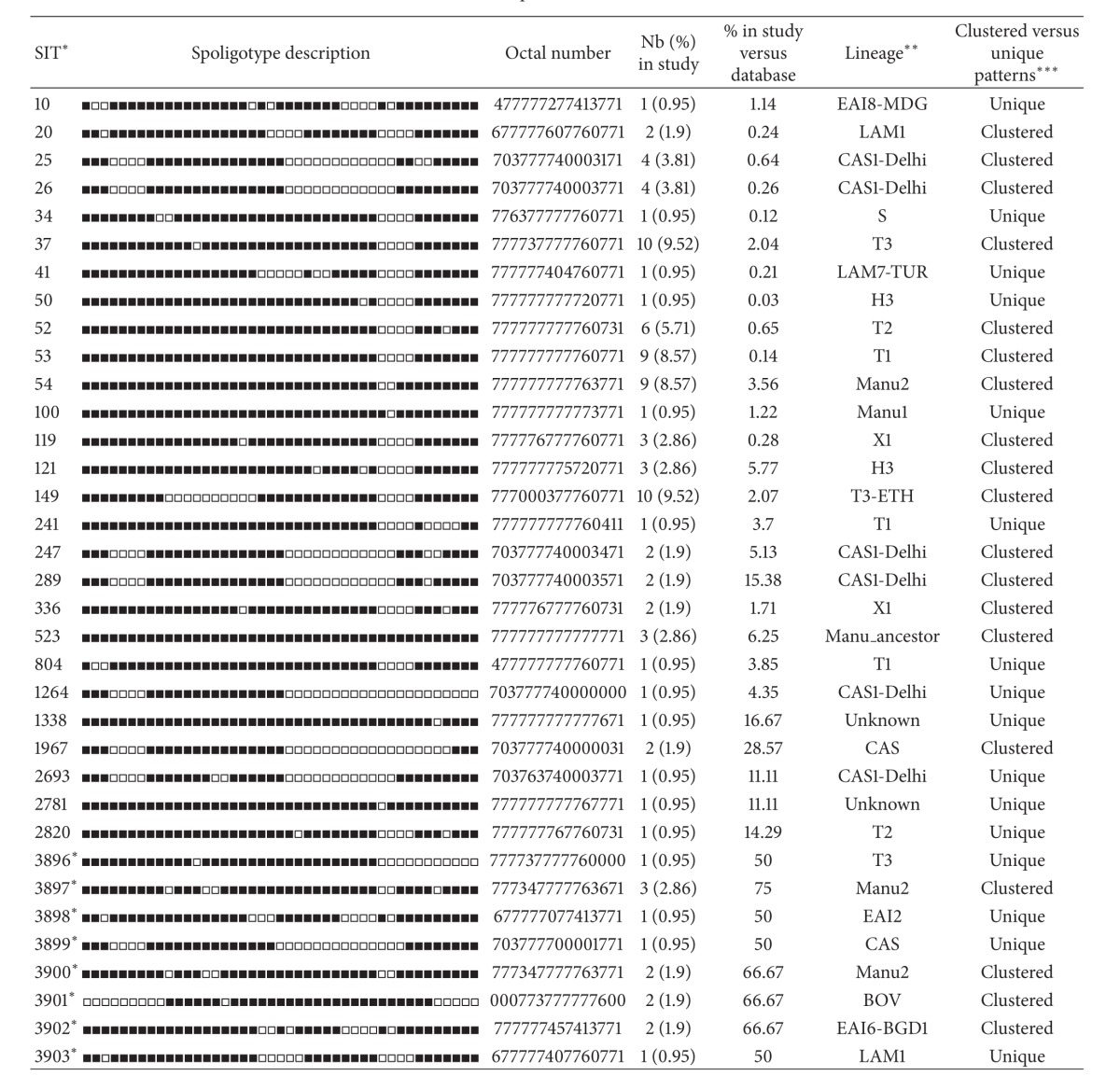

*A total of 27/35 SITs containing 83 isolates matched a preexisting shared-type in the database, whereas 8/35 SITs (*n* = 13 isolates) were newly created. A total of 19/35 SITs containing 80 isolates were clustered within this study (2 to 10 isolates per cluster) while 16/35 SITs containing 16 strains were unique (for total unique strains, one should add to this number the 9 orphan strains which brings the number of unclustered isolates in this study to 25/105 (23.81%) and clustered isolates to 80/105 (76.19%)). Note that SITs followed by an asterisk indicates “newly created” SITs due to 2 or more strains belonging to an identical new pattern within this study or after a match with an orphan in the database; SIT designations followed by number of strains: 3896* this study *n* = 1. DEU *n* = 1; 3897* this study *n* = 3. USA *n* = 1; 3898* this study *n* = 1. AUS *n* = 1; 3899* this study *n* = 1. NPL *n* = 1; 3900* this study *n* = 2. CHN *n* = 1; 3901* this study *n* = 2. FXX *n* = 1; 3902* this study *n* = 2. MYS *n* = 1; 3903* this study *n* = 1. BRA *n* = 1.

**Lineage designations according to SITVIT2 using revised SpolDB4 rules; “unknown” designates patterns with signatures that do not belong to any of the major lineages described in the database.

***Clustered strains correspond to a similar spoligotype pattern shared by 2 or more strains “within this study”; as opposed to unique strains harboring a spoligotype pattern that does not match with another strain from this study. Unique strains matching a preexisting pattern in the SITVIT2 database are classified as SITs, whereas in case of no match, they are designated as “orphan” (see Table 1).

**Table 3:** Description of clusters containing 3 or more isolates in this study and their worldwide distribution in the SITVIT2 database.

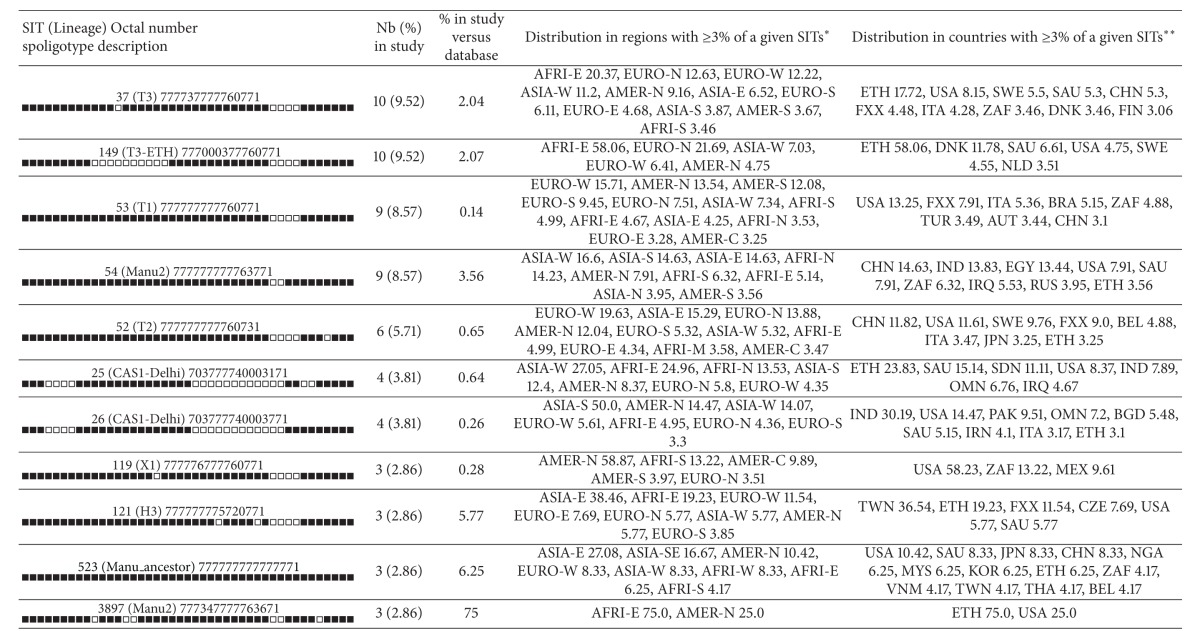

*Worldwide distribution (analysis made on August 18, 2013) is reported for regions with more than 3% of given SITs as compared to their total number in the SITVIT2 database. The definition of macrogeographical regions and subregions (http://unstats.un.org/unsd/methods/m49/m49regin.htm) is according to the United Nations; regions: AFRI (Africa), AMER (Americas), ASIA (Asia), EURO (Europe), and OCE (Oceania), subdivided in: E (Eastern), M (Middle), C (Central), N (Northern), S (Southern), SE (South-Eastern), and W (Western). Furthermore, CARIB (Caribbean) belongs to Americas, while Oceania is subdivided in 4 subregions, AUST (Australasia), MEL (Melanesia), MIC (Micronesia), and POLY (Polynesia). Note that in our classification scheme, Russia has been attributed a new subregion by itself (Northern Asia) instead of including it among the rest of Eastern Europe. It reflects its geographical localization as well as due to the similarity of specific TB genotypes circulating in Russia (a majority of Beijing genotypes) with those prevalent in Central, Eastern, and Southeastern Asia.

**The 3-letter country codes are according to http://en.wikipedia.org/wiki/ISO_3166-1_alpha-3; countrywide distribution is only shown for SITs with ≥3% of given SITs as compared to their total number in the SITVIT2 database.

**Table 4:** Distribution in Ethiopia and neighboring subregions of 11 predominant spoligotype patterns in this study (clusters containing 3 or more isolates), as seen through the updated SITVIT2 database^a^.

Octal code (SIT) lineage	Ethiopia		*P* value	Distribution in five African subregions and neighboring Western Asia in SITVIT2^b^											
	This study	SITVIT2		AFRI-E	*P*-value	AFRI-N	*P* value	AFRI-W	*P* value	AFRI-M	*P* value	AFRI-S	*P* value	ASIA-W	*P* value
	*N*/*t* (%)	*N*/*t* (%)		*N*/*t* (%)		*N*/*t* (%)		*N*/*t* (%)		*N*/*t* (%)		*N*/*t* (%)		*N*/*t* (%)	
37 (T3)	10/105	77/1507	0.072	90/4138	0.0003*	7/2145	<0.0001*	6/1985	<0.0001*	0/808	<0.0001*	17/4580	<0.0001*	55/4790	<0.0001*
777737777760771	(9.52)	(5.11)		(2.17)		(0.33)		(0.30)		(0.00)		(0.37)		(1.15)	
149 (T3-ETH)	10/105	271/1507	0.056	271/4138	0.26	1/2145	<0.0001*	0/1985	<0.0001*	0/808	<0.0001*	1/4580	<0.0001*	34/4790	<0.0001*
777000377760771	(9.52)	(17.98)		(6.55)		(0.05)		(0.00)		(0.00)		(0.02)		(0.71)	
53 (T1)	9/105	154/1507	0.62	283/4138	0.52	221/2145	0.60	109/1985	0.21	24/808	0.006*	312/4580	0.51	459/4790	0.75
777777777760771	(8.57)	(10.22)		(6.84)		(10.30)		(5.49)		(2.97)		(6.81)		(9.58)	
54 (Manu2)	9/105	0/1507	<0.0001*	4/4138	<0.0001*	36/2145	<0.0001*	3/1985	<0.0001*	0/808	<0.0001*	16/4580	<0.0001*	42/4790	<0.0001*
777777777763771	(8.57)	(0.00)		(0.10)		(1.68)		(0.15)		(0.00)		(0.35)		(0.88)	
52 (T2)	6/105	24/1507	0.0036*	40/4138	0.001*	6/2145	<0.0001*	6/1985	<0.0001*	33/808	0.44	21/4580	<0.0001*	49/4790	0.0013*
777777777760731	(5.71)	(1.59)		(0.97)		(0.28)		(0.30)		(4.08)		(0.46)		(1.02)	
25 (CAS1-Delhi)	4/105	144/1507	0.066	151/4138	0.79	84/2145	1.0	2/1985	<0.0001*	1/808	0.0009*	3/4580	<0.0001*	168/4790	0.79
703777740003171	(3.81)	(9.56)		(3.65)		(3.92)		(0.10)		(0.12)		(0.07)		(3.51)	
26 (CAS1-Delhi)	4/105	43/1507	0.55	71/4138	0.12	16/2145	0.013*	0/1985	<0.0001*	0/808	0.0002*	6/4580	<0.0001*	213/4790	1.0
703777740003771	(3.81)	(2.85)		(1.72)		(0.75)		(0.00)		(0.00)		(0.13)		(4.45)	
119 (X1)	3/105	1/1507	0.001*	4/4138	0.0005*	5/2145	0.005*	2/1985	0.0012*	0/808	0.0016*	143/4580	1.0	9/4790	0.002*
777776777760771	(2.86)	(0.07)		(0.10)		(0.23)		(0.10)		(0.00)		(3.12)		(0.19)	
121 (H3)	3/105	7/1507	0.024*	7/4138	0.002*	0/2145	0.0001*	0/1985	0.0001*	0/808	0.0016*	0/4580	<0.0001*	3/4790	0.0002*
777777775720771	(2.86)	(0.46)		(0.17)		(0.00)		(0.00)		(0.00)		(0.00)		(0.06)	
523 (Manu_ancestor)	3/105	0/1507	0.0003*	0/4138	<0.0001*	1/2145	0.0004*	4/1985	0.004*	0/808	0.0016*	2/4580	0.0001*	4/4790	0.0003*
777777777777771	(2.86)	(0.00)		(0.00)		(0.05)		(0.20)		(0.00)		(0.04)		(0.08)	
3897 (Manu2)	3/105	0/1507	0.0003*	0/4138	<0.0001*	0/2145	0.0001*	0/1985	0.0001*	0/808	0.0016*	0/4580	<0.0001*	0/4790	<0.0001*
777347777763671	(2.86)	(0.00)		(0.00)		(0.00)		(0.00)		(0.00)		(0.00)		(0.00)	

^a^Asterisk denotes statistically significant differences (*P* < 0.05) as compared to the total number of strains for a given pattern in this study (*n* = 105 strains). For statistical comparison of differences observed, note that Pearson's Chi-square test was used when more than 80% of data had an expected value greater than 5, and Fisher's Exact Test for remaining data with smaller values (at least 20% of data having values less than 5).

^b^The definition of subregions is according to the United Nations (http://unstats.un.org/unsd/methods/m49/m49regin.htm). The table summarizes distribution in this study (*n* = 105) versus all strains reported from Ethiopia in the international database (*n* = 1507, excluding the present study), the five African subregions (*n* = 13656), and neighboring Western Asian countries (ASIA-W, *n* = 4790). AFRI-E: Burundi, Comoros, Ethiopia, Kenya, Madagascar, Malawi, Mauritius, Mozambique, Reunion, Tanzania, Uganda, Zambia, and Zimbabwe; AFRI-N: Algeria, Egypt, Libyan Arab Jamahiriya, Morocco, Sudan, and Tunisia; AFRI-W: Benin, Burkina Faso, Gambia, Guinea, Guinea-Bissau, Ivory Coast, Mali, Mauritania, Nigeria, Senegal, and Sierra Leone; AFRI-M: Cameroon, Central African Republic, Chad, and Democratic Republic of Congo; AFRI-S: Botswana, Namibia, South Africa, and Swaziland; ASIA-W: Armenia, Azerbaijan, Georgia, Iraq, Israel and Palestinian territories, Oman, Saudi Arabia, Turkey, and Yemen (SITVIT2 database comparison made on August 21st 2013 by David Couvin and Nalin Rastogi).
